# LapCR-Net: A Lightweight Monocular Depth Estimation Network via Laplacian Residual Reconstruction

**DOI:** 10.3390/s26154912

**Published:** 2026-08-04

**Authors:** Linghao Li, Yingjun Zhao, Kai Qin, Donghua Lu, Huilin Yang, Ximin Wang

**Affiliations:** 1 Beijing Research Institute of Uranium Geology, Beijing 100029, China; lilinghao@briug.cn (L.L.); qinkai@briug.cn (K.Q.); ludonghua@briug.cn (D.L.); yanghuilin@briug.cn (H.Y.); wangximin@briug.cn (X.W.); 2 National Key Laboratory of Uranium Resources Exploration-Mining and Nuclear Remote Sensing, Beijing 100029, China

**Keywords:** monocular depth estimation, lightweight network, Laplacian residual reconstruction, residual learning, uncertainty estimation

## Abstract

**Highlights:**

**What are the main findings?**

- A lightweight monocular depth estimation network (LapCR-Net) is proposed, integrating Laplacian residual reconstruction with a collaborative mechanism between the Structure-aware Feature Recalibration (SFR) module and the Depth-guided Convolution Module (DCM) to enhance structural consistency and detail recovery.
- The method achieves competitive accuracy on both indoor and outdoor benchmarks with only 5.4 M parameters, demonstrating strong parameter efficiency under real-time constraints.

**What are the implications of the main findings?**

- The proposed Laplacian residual reconstruction framework provides an effective solution to alleviate cross-scale inconsistency and over-smoothing in lightweight depth estimation models.
- The model offers practical potential for deployment in resource-constrained scenarios, such as mobile platforms and autonomous driving systems.

**Abstract:**

Monocular depth estimation plays a crucial role in applications such as autonomous driving and mobile 3D reconstruction. However, existing lightweight methods are often constrained by limited computational resources and rely on shallow feature representations for direct depth regression. As a result, cross-scale residual information is insufficiently modeled, which limits their ability to preserve structural consistency and recover fine-grained details in complex scenes. To address these challenges, we propose LapCR-Net, a lightweight monocular depth estimation network based on Laplacian residual reconstruction. Specifically, we formulate a progressive Laplacian residual framework that decomposes depth prediction into a coarse-to-fine multi-scale refinement process. To enhance feature representation in the decoder, we introduce a Structure-aware Feature Recalibration (SFR) module and a Depth-guided Convolution Module (DCM), which strengthen spatial semantic correlations and improve residual prediction across scales. Furthermore, we design an uncertainty-driven collaborative refinement strategy to adaptively adjust residual correction strength. By estimating prediction uncertainty, the proposed strategy sharpens object boundaries while suppressing texture artifacts. Extensive experiments on the NYU-Depth V2 and KITTI benchmarks demonstrate that LapCR-Net achieves competitive performance with only 5.4 M parameters. In particular, it shows clear advantages in structural preservation and detail reconstruction, achieving a favorable trade-off between accuracy and computational efficiency.

## 1. Introduction

Monocular Depth Estimation (MDE) aims to predict per-pixel depth from a single two-dimensional (2D) RGB image and serves as a fundamental technique for 3D geometric scene perception [1]. By requiring only a single camera to estimate scene depth, MDE offers significant advantages in terms of hardware cost, system complexity, and deployment flexibility. Consequently, it has been widely applied in fields such as robot navigation, autonomous driving, and 3D reconstruction [2,3,4]. However, inferring 3D depth from 2D visual information is inherently ill-posed, as it is highly susceptible to factors such as scale ambiguity, texture deficiency, and illumination variations [1,5]. In particular, under resource-constrained scenarios such as mobile platforms, achieving accurate depth recovery and reliable scene perception while maintaining real-time performance remains a significant challenge.

With the rapid advancement of deep learning, data-driven monocular depth estimation methods have achieved significant progress in both feature representation and prediction accuracy [6]. Benefiting from the strengths of convolutional neural networks (CNNs) in local feature extraction and efficient inference, as well as the capability of Vision Transformers (ViTs) in modeling long-range dependencies and global contextual information, deep learning has become the dominant paradigm in this field [7,8]. Most existing approaches are built upon the encoder–decoder (ED) framework, in which the encoder progressively extracts multi-scale semantic features, while the decoder restores spatial resolution to reconstruct the depth map. Owing to its structural simplicity and training stability, the ED architecture has become the mainstream paradigm for monocular depth estimation. In this framework, the encoder captures hierarchical feature representations through successive downsampling, while the decoder integrates shallow spatial details and deep semantic information via skip connections, thereby improving depth prediction accuracy and boundary recovery. Within this paradigm, recent studies have primarily focused on feature modeling, cross-scale interaction, and decoding reconstruction strategies, achieving notable improvements in structural representation and fine-grained detail recovery [9,10].

Although existing lightweight networks effectively reduce model complexity through depthwise separable convolutions, the effective receptive field (ERF) remains insufficient to capture complex spatial structural relationships in challenging scenes [11]. Early representative works, such as MobileNet [12], ShuffleNet [13], and FastDepth [14], achieve efficient inference through depthwise separable convolutions; however, depthwise convolutions inherently weaken inter-channel information interaction. To address this limitation, hybrid architectures combining CNNs and Transformers have emerged as a promising direction. For instance, Lite-Mono [15] introduces global context modeling, but it primarily operates on deep features. As a result, low-level features remain constrained by local receptive fields, leading to discontinuities in semantic representation. Lightweight architectures such as MobileViT [16] and EdgeNeXt [17] mainly rely on simple serial stacking of modules, which limits effective cross-layer feature interaction. Moreover, the MsGf framework proposed by Tian et al. [18] enhances multi-scale responses through cross-dimensional feature extraction, but its design primarily focuses on local parallel fusion and remains limited in modeling cross-layer semantic alignment. Therefore, it is essential to develop a feature refinement mechanism that enhances geometric structure perception while facilitating cross-layer feature fusion under low computational overhead.

In addition to the limitations in feature extraction, lightweight monocular depth estimation models commonly adopt unstructured upsampling strategies in the decoding stage, which limit the recovery of high-frequency details and the correction of prediction errors. Representative lightweight networks such as FastDepth [13] and GuideDepth [19] typically adopt U-Net-style decoders, restoring spatial resolution via bilinear interpolation or transposed convolution, which tend to oversmooth sharp edges. To alleviate these issues, some studies attempt to improve detail recovery by enhancing skip connections or introducing multi-scale feature modulation. For example, Depth Anything [20] achieves high-quality depth reconstruction with a DPT decoder, but the heavy computational cost of large-scale feature reassembly limits its deployment on embedded devices. Similarly, Song et al. [21] introduced a Laplacian pyramid-based decoder that progressively predicts multi-scale depth residuals and reconstructs the final depth map through coarse-to-fine additive synthesis. However, their residual prediction is mainly guided by decoder features and explicit RGB Laplacian components, without explicitly using the reconstructed coarse depth as a spatial prior or modeling pixel-wise prediction reliability.

Although various methods have been proposed to alleviate the above issues, most of them focus on improving a single component and lack a unified optimization strategy. In terms of encoder design, Hwang et al. [22] combine CNNs with Transformers to enhance global interaction, but fail to address the inherent receptive field discrepancies across multi-scale features. Similarly, architectures such as MonoViT [23] and DepthFormer [24] introduce global context modeling, yet still suffer from semantic discontinuities and computational redundancy when handling multi-scale features. For decoder and reconstruction strategies, BinsFormer [25] improves depth representation by predicting depth distributions via a Transformer decoder, but remains limited in mitigating cross-layer semantic gaps. Methods such as HR-Depth [26] and R-MSFM [27] enhance local prediction through improved cross-layer feature propagation, yet their upsampling processes lack explicit structural constraints, resulting in insufficient cross-layer semantic alignment. Overall, most existing lightweight MDE methods optimize encoder feature extraction and decoder reconstruction separately, while the interaction between cross-scale semantic inconsistency and structural over-smoothing remains insufficiently explored. Therefore, a unified lightweight framework is needed to jointly enhance multi-scale feature representation, constrain the upsampling process, and adaptively correct high-frequency structural residuals.

To address these challenges, we propose LapCR-Net, a lightweight MDE framework based on Laplacian residual reconstruction. LapCR-Net targets cross-scale inconsistency and structural over-smoothing in lightweight MDE by decomposing depth recovery into low-frequency structure prediction and high-frequency residual correction within a unified framework. Through the collaboration of the SFR, DCM, and uncertainty-aware refinement modules, the proposed framework enables structure-aware, depth-guided, and adaptive residual reconstruction. The main contributions of this work are summarized as follows:

(1) We propose a collaborative structure–depth feature refinement mechanism to enhance lightweight MDE representation. Unlike conventional methods that separately enhance local or global features, the proposed mechanism jointly models structural features, depth-guided residuals, and global contextual dependencies within a unified framework. Specifically, the Structure-aware Feature Recalibration (SFR) module enhances geometry-sensitive features with negligible computational overhead, while the Depth-guided Convolution Module (DCM) exploits previous-scale depth priors to guide cross-layer semantic alignment and high-frequency detail reconstruction. Together with the bottleneck Triple Cross-Attention (TCA) module, this collaborative design alleviates structural blur and semantic inconsistency, establishing a unified structure–depth representation for progressive Laplacian residual reconstruction.

(2) We propose a depth-guided progressive Laplacian residual reconstruction framework for structured depth decoding. Unlike existing Laplacian pyramid-based depth residual decoders that combine explicit RGB Laplacian components with decoder features and propagated residual information, the proposed framework uses the reconstructed depth from the preceding scale as an explicit spatial prior to guide current-scale residual prediction. Specifically, depth prediction is decomposed into low-frequency base estimation and high-frequency Laplacian residual correction, while a learned upsampling strategy models upsampling as a residual compensation process (${depth}_{up}+\delta$). This structured design explicitly constrains cross-scale reconstruction, reducing error accumulation while improving structural consistency and boundary-region accuracy.

(3) We propose an uncertainty-driven edge-guided collaborative refinement strategy for adaptive residual reconstruction. Unlike conventional refinement methods that apply fixed correction across all pixels, the proposed strategy estimates pixel-wise uncertainty and incorporates it into the residual reconstruction process. Specifically, a Sigma Head predicts uncertainty maps, which are jointly exploited with image edge information to guide adaptive residual correction. This uncertainty-aware design dynamically modulates residual correction according to prediction confidence, enhancing structural details in reliable regions while suppressing error propagation in uncertain areas.

This work adopts an improved U-shaped encoder–decoder architecture, featuring an encoder built upon a modified MobileNetV3 backbone to enhance multi-scale feature representation while maintaining low computational overhead. Experimental results demonstrate that the proposed method achieves competitive depth estimation accuracy with real-time inference efficiency. In particular, it shows strong capability in preserving structural consistency and depth continuity, providing reliable depth perception for resource-constrained applications such as 3D reconstruction and autonomous driving.

## 2. Materials and Methods

### 2.1. Overall Framework of LapCR-Net

**Figure 1:**
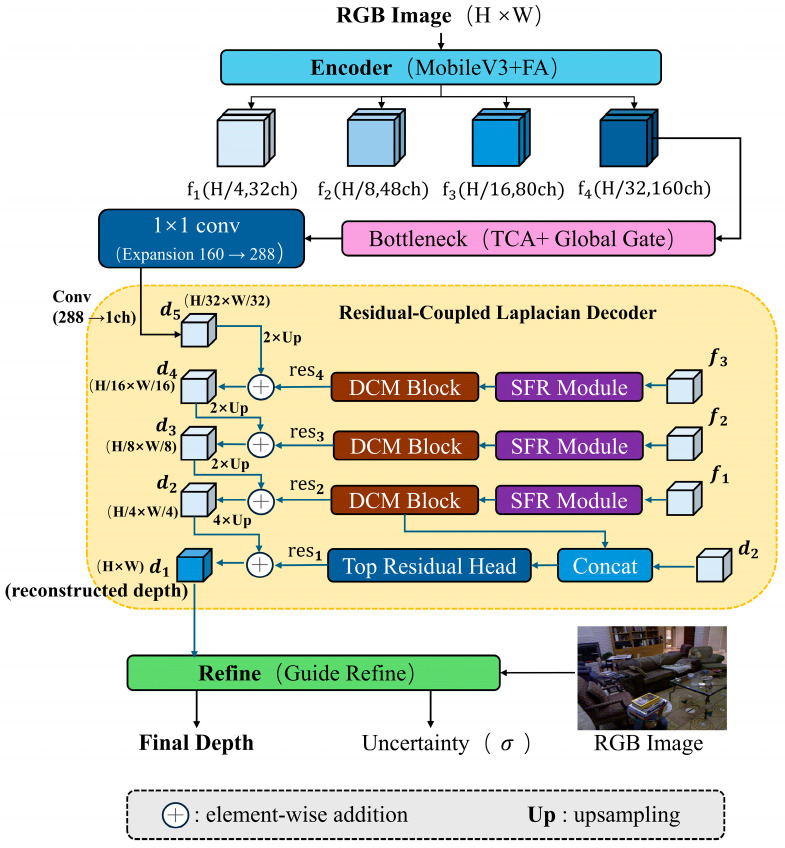
Overall Framework of LapCR-Net.

The overall framework of LapCR-Net adopts a U-shaped encoder–decoder structure, as shown in Figure 1. By combining feature-enhanced encoding with explicit Laplacian residual reconstruction, the network achieves progressive depth refinement and improved prediction quality.

In Figure 1, $d_{5}$–$d_{1}$ denote the multi-scale depth predictions in the progressive Laplacian residual reconstruction process. The labels “2× Up” and “4× Up” indicate the upsampling operations between adjacent depth scales. Specifically, $d_{5}$, $d_{4}$, $d_{3}$, $d_{2}$, and $d_{1}$ correspond to H/32×W/32, H/16×W/16, H/8×W/8, H/4×W/4, and H×W, respectively. The predictions from $d_{5}$ to $d_{2}$ are progressively refined through 2× upsampling and residual correction, while $d_{2}$ is further upsampled by 4× to obtain the full-resolution depth $d_{1}$.

The encoder, designated as LapCR-Encoder, is constructed upon the MobileNetV3 backbone. A Feature Alignment (FA) module is introduced to fuse shallow texture details with deep semantic information, thereby enhancing multi-scale feature representation. At the bottleneck, a module composed of Triple Cross-Attention (TCA) and a Global Gate (GG) mechanism is designed to expand the receptive field and establish long-range spatial and channel dependencies. This module provides refined global context to guide the subsequent decoding process.

The decoder, designated as LapCR-Decoder, is constructed based on a progressive Laplacian residual reconstruction framework, enabling coarse-to-fine depth recovery through hierarchical residual refinement. The SFR module enhances feature representation and guides residual prediction, while the DCM generates and integrates Laplacian residuals under the structural constraints provided by the previous-scale depth, thereby enabling effective cross-scale information fusion. Combined with a top-level residual head, the depth prediction is progressively refined across scales. This decoding strategy preserves global geometric consistency while effectively improving the recovery of local boundaries and high-frequency details.

### 2.2. MobileNetV3 Backbone

MobileNetV3 is a lightweight convolutional neural network optimized via hardware-aware neural architecture search (NAS) and the NetAdapt algorithm, aiming to achieve an optimal trade-off between inference latency and accuracy on mobile devices [28]. Unlike conventional networks, MobileNetV3 adapts its depth and convolutional configurations to specific hardware platforms, leading to improved feature extraction efficiency. Therefore, MobileNetV3-Large is adopted as the backbone encoder of LapCR-Net to enable effective feature encoding under limited computational resources.

**Figure 2:**
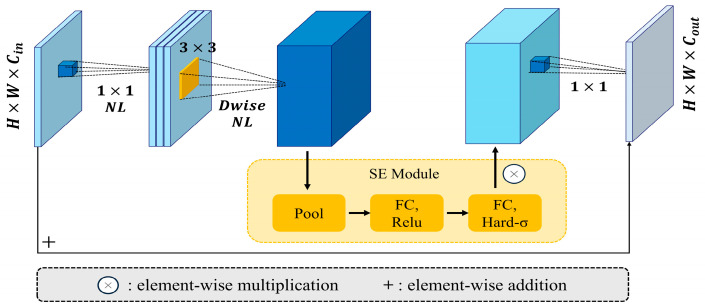
Structure of the MobileNetV3 inverted residual block with SE attention mechanism [30].

The core building block of the network is the inverted residual block incorporating a lightweight attention mechanism. As illustrated in Figure 2, the module first expands the feature dimension through a pointwise convolution, followed by a depthwise convolution for spatial feature extraction. A squeeze-and-excitation (SE) module is then introduced to perform dynamic channel-wise recalibration. Finally, a projection layer is applied to reduce the feature dimension. Furthermore, the network adopts h-swish and hard-sigmoid activation functions instead of conventional nonlinear functions, reducing computational complexity while maintaining representation capability [29].

Although MobileNetV3 demonstrates strong performance in image classification, its direct application to pixel-wise depth estimation reveals inherent limitations. Continuous downsampling operations enlarge the receptive field but inevitably reduce spatial resolution, leading to the loss of spatial geometric structures and high-frequency texture details. Moreover, the original SE module primarily responds to global statistical features [31], limiting its effectiveness in capturing fine-grained local boundary variations that are critical for depth estimation. To address these limitations, we introduce structure-aware and cross-scale collaborative mechanisms based on the backbone architecture to improve its adaptability for dense depth prediction tasks.

### 2.3. LapCR-Net Architecture

#### 2.3.1. Encoder Design (LapCR-Encoder)

As the front-end module of a monocular depth estimation network, the encoder is responsible for extracting multi-scale features from raw RGB images that are both semantically rich and geometrically discriminative [32]. To address the challenges of large-scale variations, uneven semantic distribution, and the loss of boundary details in monocular depth estimation, we design a lightweight LapCR-Encoder with global modeling capability.

The LapCR-Encoder adopts a hierarchical feature extraction strategy, consisting of a multi-scale feature enhancement module and a bottleneck feature modeling module. Built upon the MobileNetV3 backbone, the encoder progressively downsamples the input RGB image to extract features at multiple spatial resolutions. A Feature Alignment (FA) module is introduced at each scale to fuse shallow texture details with deep semantic information. At the bottleneck, a module composed of Triple Cross-Attention (TCA) and a Global Gate (GG) mechanism performs global modeling on high-level features and generates coarse-scale depth priors to guide the subsequent decoding stage.

Based on the above design, the LapCR-Encoder effectively preserves multi-scale spatial structural information while capturing long-range spatial and channel dependencies. It further provides refined and stable global context to guide the subsequent Laplacian residual reconstruction in the decoder.

#### 2.3.2. Decoder Design (LapCR-Decoder)

As the backend module of a monocular depth estimation network, the decoder is responsible for progressively reconstructing a dense depth map from the encoded features at the original image resolution [32]. To address issues such as edge detail blurring during upsampling and the accumulation of residual prediction errors, we design a Laplacian residual coupling reconstruction-based decoder, termed LapCR-Decoder.

The LapCR-Decoder adopts a coarse-to-fine progressive depth reconstruction strategy with layer-wise refinement, where the core process is collaboratively accomplished by a Structure-aware Feature Recalibration (SFR) module and a Depth-guided Convolution Module (DCM).

During the decoding process, the decoder takes the global context feature $X_{\mathrm{out}}$ from the encoder bottleneck as the starting point, and generates an initial coarse depth map through a linear projection, providing a global structural reference for subsequent hierarchical reconstruction.

During the hierarchical upsampling stage, the decoder fuses multi-scale enhanced features $\left\{f_{i}\right\}$ from the encoder via skip connections. The SFR module is employed to perform structure-aware recalibration on these guided features, thereby enhancing their capability to represent scene geometry and object boundaries, which provides stable structural constraints for residual prediction. Building upon this, the DCM module—guided by the depth maps from the preceding scale—generates Laplacian residuals through depth-guided convolutions. These residuals are then coupled with the upsampled depth to achieve progressive compensation and refinement. By fully leveraging global geometric priors, this architecture effectively restores boundary details and high-frequency components while alleviating scale ambiguity and structural over-smoothing in monocular depth estimation.

### 2.4. Core Modules and Mechanisms

#### 2.4.1. FA-Mobile Feature Enhancement Module

**Figure 3:**
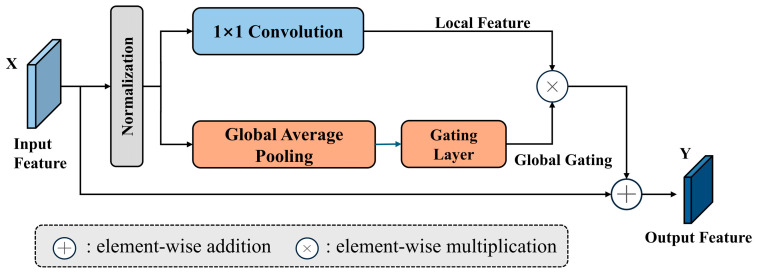
FA-Mobile feature enhancement module.

MobileNetV3 shows limited representation capability in complex scenes, particularly when handling multi-scale features, where performance bottlenecks may arise. To address this issue, we introduce a Feature Augmentation for Mobile (FA-Mobile) module into the multi-scale feature layers of the backbone to enhance the encoder’s ability to capture multi-scale structures. The architecture of the FA-Mobile module is illustrated in Figure 3.

The input RGB image is processed by the MobileNetV3 feature extractor to produce four feature maps at different resolutions. The first feature map (Layer1) has a spatial resolution of 1/2 of the input image. Each subsequent layer reduces the resolution by a factor of two, resulting in Layer2, Layer3, and the smallest Layer4, which has a resolution of 1/32 of the original image. Through this hierarchical extraction strategy, the network progressively reduces spatial resolution while capturing increasingly abstract semantic information.

The FA-Mobile feature enhancement module adopts a dual-branch design to enhance shallow semantic representation while preserving fine-grained structural details. Let the input feature at a given scale be denoted as $X\in R^{C\times H\times W}$. The module first applies normalization to mitigate distribution shifts across different scales, ensuring stability during feature fusion. Subsequently, the enhancement is then performed by parallel local and global branches. The local branch employs a lightweight pointwise convolution mapping $\phi_{l}(\cdot )$ to reorganize and enhance channel features, producing a local response ${L=\phi }_{l}(X)$. In parallel, the global branch extracts channel-wise semantics via global average pooling (GAP), followed by a mapping function G to generate a global semantic gate G, defined as ${G=\phi }_{g}(GAP(X))$.

During the feature fusion stage, the FA-Mobile module adopts an adaptive modulation strategy to perform channel-wise multiplication between the local response L and the global gating G, enabling the local features to be dynamically enhanced under the guidance of global semantics. The final output feature Y is constructed in the form of a residual connection, as shown in Equation (1).

(1) Y=X+L⊙G

where ⊙ denotes channel-wise multiplication.

This dual-branch synergistic mechanism enhances the edge and texture representation of shallow features while effectively mitigating the over-smoothing of deep features during propagation under the guidance of global information. With minimal computational overhead, the FA-Mobile module achieves efficient cross-scale feature augmentation, thereby providing a stable feature foundation for the subsequent Laplacian residual reconstruction decoding process.

#### 2.4.2. Global Context Modeling at the Bottleneck Layer

In monocular depth estimation, the encoder bottleneck layer is characterized by low spatial resolution and a large receptive field, playing a crucial role in capturing the overall scene structure and geometric layout. Although the deep features have been enhanced by the FA-Mobile module and exhibit strong semantic representation capability, their ability to model long-range spatial dependencies remains limited due to the reduced spatial resolution.

**Figure 4:**
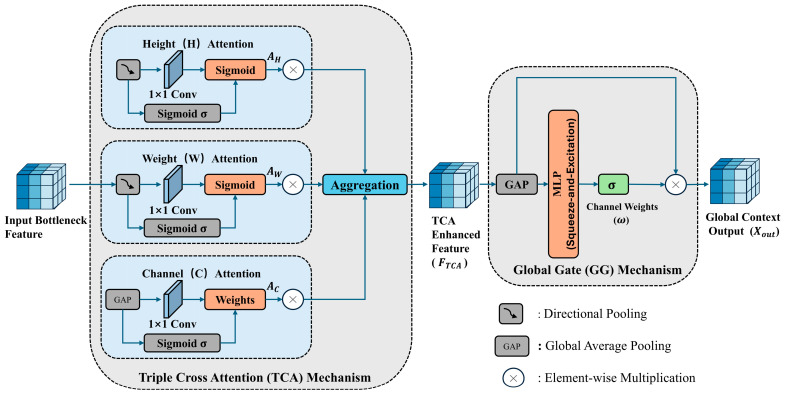
Schematic diagram of the Triple Cross-Attention (TCA) module architecture.

To address this issue, the LapCR-Encoder introduces a global context modeling module at the bottleneck layer, which performs feature modeling and channel-wise recalibration on the deepest-scale features via Triple Cross-Attention (TCA) and a Global Gate (GG) mechanism. By establishing long-range dependencies across both spatial and channel dimensions, the module enhances global semantic information related to the scene geometry while suppressing irrelevant background interference. The detailed architecture of the module is illustrated in Figure 4.

The TCA module jointly models feature representations from three orthogonal dimensions, namely horizontal (H), vertical (W), and channel (C). By leveraging multi-dimensional attention, it captures long-range dependencies within the features, enabling the network to obtain global spatial structure and channel-wise semantic distributions with relatively low computational complexity.

During the processing, the module first extracts salient information from different projection planes through cross-dimensional pooling and dimensional mapping. Taking the height–channel plane as an example, the attention weight $A_{H}$ is computed as:

(2) $$A_{H}=\sigma (\phi_{H}({Pool}_{w}(f_{4})))$$

where the deepest feature output from the encoder is denoted as $f_{4}\in R^{C_{4}\times \frac{H}{32}\times \frac{W}{32}}$, ${\mathrm{Pool}}_{w}$ represents the global pooling operator along the width dimension, $\phi_{H}$ denotes a 1×1 convolution mapping, and σ is a nonlinear activation function.

Similarly, the attention weights along the width and channel dimensions, denoted as $A_{W}$ and $A_{C}$, can be obtained in the same manner. The aggregation operation is performed by learnable weighted fusion. Specifically, the three attention branches first generate attention-enhanced responses from the horizontal, vertical, and channel dimensions, denoted as $F_{H}$, $F_{W}$, and $F_{C}$, respectively:

(3) $$F_{H}=f_{4}⊙A_{H},F_{W}=f_{4}⊙A_{W},F_{C}=f_{4}⊙A_{C}$$

Instead of directly concatenating these responses, TCA integrates them using learnable coefficients:

(4) $$F_{TCA}=\alpha_{H}F_{H}+\alpha_{W}F_{W}+\alpha_{C}F_{C}$$

where $\alpha_{H}$, $\alpha_{W}$, and $\alpha_{C}$ are learnable parameters optimized during training. This strategy enables the network to adaptively balance directional spatial dependencies and channel-wise semantic information according to different scene structures. The aggregated feature $F_{TCA}$ is then used as the final attention-enhanced representation, which simultaneously encodes rich semantic information and geometric structural information.

Based on multi-dimensional feature modeling, the module further introduces a Global Gate mechanism to perform channel-wise recalibration. Specifically, global average pooling is first applied to extract a global semantic descriptor $\upsilon \in R^{C}$. Subsequently, a gating mapping function is constructed via two lightweight fully connected layers to generate channel-wise modulation weights ω, as formulated in Equation (5).

(5) $$\omega =\sigma \left(W_{2}\cdot \delta \left(W_{1}\cdot v\right)\right)$$

where δ denotes the ReLU activation function, and $W_{1}$ and $W_{2}$ are learnable weight matrices. Finally, the gating weights ω are applied to $F_{\mathrm{TCA}}$, and the refined feature $X_{\mathrm{out}}$ is obtained via channel-wise multiplication, as formulated in Equation (6).

(6) $$X_{out}=\omega ⊗F_{TCA}$$

Through the synergy of the TCA and GG modules, the bottleneck feature $X_{\mathrm{out}}$ establishes long-range spatial dependencies among pixels while achieving semantic focus along the channel dimension. This global context constraint mechanism enables the encoder to form high-level representations with strong geometric consistency at the bottleneck layer, thereby providing a stable structural prior for the progressive residual reconstruction in the subsequent LapCR-Decoder.

#### 2.4.3. Residual-Coupled Laplacian Reconstruction Mechanism

The LapCR-Decoder draws inspiration from the hierarchical reconstruction paradigm of the Laplacian pyramid [33,34], reformulating depth estimation as a progressive reconstruction problem of multi-scale Laplacian residuals. By modeling the geometric differences between adjacent depth scales, the network enables layer-wise compensation of fine-grained details under the constraint of coarse-scale global structures, thereby achieving a coarse-to-fine progressive depth recovery. Compared with conventional regression-based approaches for depth map construction, this mechanism shifts the learning objective toward a more tractable local structural refinement process, effectively improving structural stability and prediction consistency across multiple scales.

Although both Song et al. [21] and LapCR-Net adopt coarse-to-fine Laplacian residual reconstruction, their residual-generation mechanisms are different. Song et al. explicitly construct an RGB Laplacian pyramid and combine the corresponding image Laplacian component with decoder features and propagated residual information at each scale. In contrast, LapCR-Net first enhances the encoder features through the SFR module and then uses the reconstructed depth from the preceding scale as an explicit spatial prior in the DCM to guide current-scale residual prediction. Furthermore, pixel-wise uncertainty is introduced to adaptively regulate the subsequent refinement. These differences enable LapCR-Net to perform structure-aware, depth-guided, and uncertainty-regulated residual prediction during progressive reconstruction.

Specifically, at the i-th decoding scale, the depth prediction $d_{i}$ is formulated as the linear combination of the upsampled depth from the previous scale and the Laplacian residual at the current scale, as defined in the following equation:

(7) $$F_{i}=SFR(f_{i})$$

(8) $$d_{i}=Up(d_{i+1})+R_{i}(F_{i},Up(d_{i+1}))$$

where Up(⋅) denotes the upsampling operator, $f_{i}$ represents the enhanced feature from the encoder at the corresponding scale, and $F_{i}$ denotes the guided feature obtained by applying the SFR module to $f_{i}$. The residual mapping $R_{i}(\cdot )$ is predicted by the DCM under the guidance of $F_{i}$ and the constraint of the upsampled depth. This residual primarily captures high-frequency geometric information at the current scale that is not represented by low-frequency structures, such as object boundaries and depth discontinuities. The generation of such residuals relies on structure-aware features and depth-guided information, the detailed implementation of which will be elaborated in Section 2.4.4 and Section 2.4.5.

This mechanism preserves global geometric consistency while progressively correcting upsampling errors through layer-wise residual refinement. As a result, it effectively suppresses error accumulation during multi-stage reconstruction and enables more accurate recovery of high-frequency structures, including boundaries and depth discontinuities.

Furthermore, an uncertainty-aware mechanism is incorporated at the decoder output to model pixel-wise prediction confidence. Specifically, an uncertainty map $\sigma_{i}$ is estimated to characterize the reliability of depth predictions across spatial locations. During the subsequent refinement process, this map is used to adaptively modulate the magnitude of residual corrections, enhancing detail recovery in high-confidence regions while suppressing error propagation in uncertain areas. By preserving global structural consistency, this mechanism effectively improves the accuracy and robustness of depth estimation in complex scenes.

#### 2.4.4. Structure-Aware Feature Recalibration Module (SFR)

**Figure 5:**
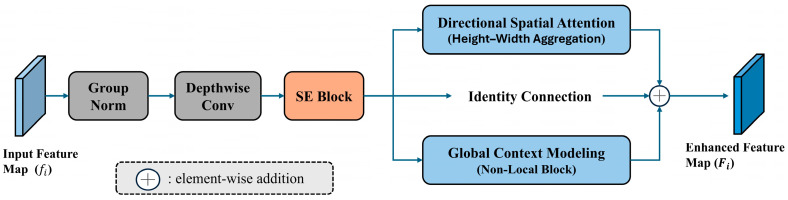
Schematic diagram of the SFR module.

In this work, a Structure-aware Feature Recalibration (SFR) module is introduced in the decoder to perform guided enhancement on the skip-connected features from the encoder, thereby providing more stable and geometrically discriminative feature representations for subsequent Laplacian residual prediction. The detailed architecture of the SFR module is illustrated in Figure 5.

The SFR module comprises three core components: local structure enhancement, channel-wise recalibration, and spatial context modeling. Specifically, the input feature $f_{i}$ undergoes normalization and depthwise separable convolution to extract local features, thereby enhancing the response to object boundaries and local geometric structures. Subsequently, a Squeeze-and-Excitation (SE) mechanism is employed to perform adaptive channel-wise recalibration, highlighting depth-relevant semantics while suppressing redundant noise. Furthermore, feature enhancement is performed through a directional spatial attention branch and a global context modeling branch. The outputs of these two branches are fused with the backbone features via residual connections, yielding the enhanced feature $F_{i}=SFR(f_{i})$, which serves as guidance for Laplacian residual prediction at the corresponding scale.

The features refined by the SFR module preserve semantic consistency while exhibiting enhanced geometric discriminability, enabling more accurate capture of subtle depth variations. This effectively alleviates scale ambiguity and structural over-smoothing caused by insufficient feature representation in monocular depth estimation.

#### 2.4.5. Depth-Guided Convolution Module (DCM)

**Figure 6:**
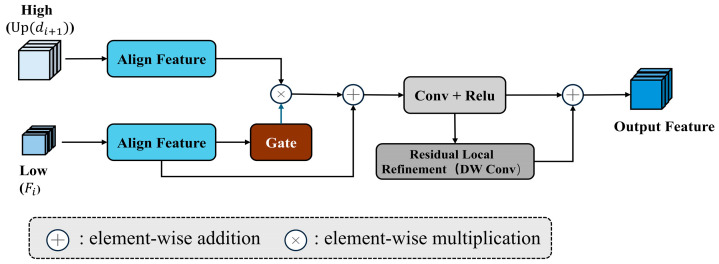
Schematic diagram of the DCM.

The DCM serves as the core prediction unit of the decoder, and its detailed architecture is illustrated in Figure 6. The module first aligns the input feature $F_{i}$ with the upsampled depth and employs a gating mechanism to achieve adaptive fusion between the features and depth priors, upon which the Laplacian residual response is generated. Subsequently, the features are updated through convolution and local refinement operations and combined with residual connections to produce the final output, thereby enabling progressive refinement of the depth at the current scale. At the end of the decoder, an uncertainty-aware mechanism [35,36] is further introduced. An independent Sigma Head is attached after the final depth prediction $d_{1}$ to estimate the uncertainty map σ. A lightweight 3×3 convolution followed by a Softplus activation is used to ensure positive uncertainty values:

(9) $$\sigma =Softplus({Conv}_{3\times 3}(d_{1}))+\varepsilon$$

where $d_{1}$ denotes the final depth prediction and ε is a small constant for numerical stability. Notably, σ does not require explicit supervision and is learned implicitly through the uncertainty-aware depth loss.

In the residual generation pathway, the DCM utilizes the coarse-scale depth as a spatial prior and reweights the feature responses through depth-guided convolution. This enables the residual prediction to focus on critical geometric transition regions, such as object boundaries. Accordingly, the Laplacian residual at the current scale, denoted as $R_{i}$, can be formulated as:

(10) $$R_{i}=H_{res}\left(F_{i}⊗G\left(Up(d_{i+1})\right)\right)$$

where G(⋅) denotes a depth-guided feature encoding operator, which projects the upsampled depth into a feature space aligned with $F_{i}$, while $H_{res}$ represents the residual regression head.

Through this design, the DCM leverages depth priors to reweight feature responses, enabling the residual prediction to focus more on critical geometric regions such as object boundaries and depth discontinuities. Compared with approaches that estimate residuals solely based on feature representations, the incorporation of depth guidance provides more stable spatial constraints during cross-scale reconstruction, thereby improving the accuracy and consistency of residual prediction. In combination with the structure-aware features provided by the SFR module, the DCM is better able to capture subtle depth variations, providing reliable residual information for subsequent Laplacian residual reconstruction.

### 2.5. Loss Function

During the training stage, a loss function framework consistent with the progressive residual architecture is designed, which mainly consists of an uncertainty-aware depth loss and an edge-aware smoothness loss.

The uncertainty-aware depth loss incorporates the uncertainty map σ predicted by the Sigma Head to adaptively weight the regression error of the final depth prediction. Specifically, the Sigma Head is attached after the final depth prediction $d_{1}$, and the uncertainty map σ is optimized jointly with the depth prediction through the uncertainty-aware loss. The uncertainty-aware depth loss is defined as:

(11) $$L_{unc}=\frac{1}{N}\sum_{p=1}^{N}\left(\frac{|d_{1}(p)\;-\;d^{gt}(p)|}{\sigma (p)}+log\;\sigma (p)\right)$$

where $d_{1}(p)$ denotes the final depth prediction at pixel p, $d^{gt}(p)$ denotes the corresponding ground-truth depth, and σ(p) denotes the pixel-wise uncertainty predicted by the Sigma Head. The uncertainty map σ is predicted rather than being provided by ground-truth supervision, and it is optimized jointly with the depth prediction through the uncertainty-aware loss. The first term serves as an error-adaptive scaling component, where the predicted uncertainty σ is used to weight the depth regression error. This allows the model to automatically reduce the influence of unreliable predictions in high-uncertainty regions, such as object boundaries, occlusions, textureless areas, and depth discontinuities, thereby improving training stability. The second term, logσ, serves as a regularization constraint to bound the magnitude of the predicted uncertainty and prevent it from increasing without limit.

Meanwhile, to preserve depth continuity and avoid over-smoothing across image edges, an edge-aware smoothness loss based on image gradients is introduced:

(12) $$L_{sm}^{\left(i\right)}=\frac{1}{N}\sum_{p=1}^{N}|\nabla d_{i}(p)|exp(-|\nabla I(p)|)$$

where $\nabla d_{i}$ and ∇I denote the spatial gradients of the predicted depth map and the input RGB image, respectively.

The uncertainty-aware depth loss is applied to the final depth prediction, while the edge-aware smoothness loss is applied to the multi-scale decoder outputs to preserve depth continuity across scales. The overall loss is defined as:

(13) $$L=L_{unc}+\alpha \sum_{i=1}^{M}\lambda_{i}L_{sm}^{\left(i\right)}$$

where M denotes the number of decoding scales, and $\lambda_{i}$ and α are balancing coefficients.

Under this loss formulation, the uncertainty-aware depth loss adaptively adjusts the regression error of the final depth prediction according to prediction reliability, improving model stability in challenging regions. The edge-aware smoothness loss, guided by image gradients, preserves depth continuity while preventing over-smoothing across boundaries. Together, these losses contribute to improved overall accuracy and robustness in depth estimation.

## 3. Results

### 3.1. Experimental Setup

#### 3.1.1. Implementation Details

The experiments are implemented using the PyTorch deep learning framework in a Python 3.9 environment. Model training and debugging are conducted on a Windows 11 system equipped with an NVIDIA GeForce RTX 3090 GPU (24 GB VRAM), with CUDA 11.8 and PyTorch 2.0.0 used for hardware acceleration. To ensure the reproducibility and consistency of the results, the trained models are evaluated on an Ubuntu 20.04 system under the same hardware configuration.

The MobileNetV3-Large encoder was initialized using ImageNet-1K-pretrained weights. All newly introduced modules were randomly initialized, and all network parameters, including those of the encoder, were optimized end-to-end.

During the training phase, an iterative training strategy with dynamic learning rate adjustment is adopted. The AdamW optimizer is employed with a weight decay coefficient of 1 × 10^−2^. A One-Cycle cosine annealing strategy is utilized, where the initial learning rate is set to 1 × 10^−4^ and gradually decays to 1 × 10^−6^ over the training process. To enhance the generalization capability of the model, data augmentation techniques are applied, including random horizontal flipping, random rotation (±5°), and color jittering in brightness, contrast, and saturation. All experiments are conducted on a single NVIDIA RTX 3090 GPU with a batch size of 16 for 50 epochs.

#### 3.1.2. Datasets

**Figure 7:**
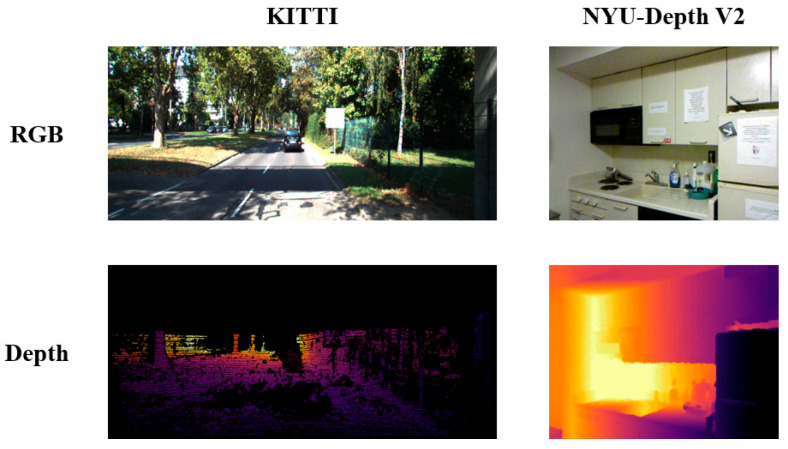
Sample images from the datasets.

In this work, experiments are conducted on two representative public benchmarks, KITTI and NYU-Depth V2, while SUN RGB-D is additionally employed for zero-shot cross-dataset evaluation. KITTI is a quintessential outdoor dataset in the computer vision community, where ground-truth depth maps are generated by aligning 64-beam LiDAR point clouds with RGB images, resulting in sparse depth labels. NYU-Depth V2 is one of the most widely used indoor benchmarks for monocular depth estimation. Collected by New York University using Microsoft Kinect sensors, it comprises a diverse range of indoor scenes with high-precision dense depth annotations. Representative examples of RGB images and their corresponding ground-truth depth maps from both datasets are shown in Figure 7.

For NYU-Depth V2, a total of 50,688 RGB–depth pairs were divided into training and validation subsets at a ratio of 90:10, while a separate test set containing 654 matched RGB–depth pairs was used exclusively for final quantitative evaluation. For KITTI, 27,052 RGB–depth pairs collected from 133 driving sequences were similarly divided into training and validation subsets at a ratio of 90:10, while a separate test set containing 1000 matched RGB–depth pairs was reserved for final evaluation. The separate test sets were not involved in model training, validation, or model selection.

For dataset-specific input preprocessing, the KITTI RGB images and depth maps were spatially resized to 640×192 pixels $\left(W\times H\right)$, whereas the NYU-Depth V2 images and depth maps were resized to 320×240 pixels $\left(W\times H\right)$. No additional cropping or padding was applied before network input. Bilinear interpolation was used for RGB images, while nearest-neighbor interpolation was applied to the depth maps to avoid introducing interpolated depth values.

To further evaluate the generalization capability of the proposed method across different data domains, SUN RGB-D was selected as an additional evaluation dataset because it differs from NYU-Depth V2 in terms of scene distribution, sensors, and acquisition conditions. All models were directly evaluated on this dataset in a zero-shot setting, without fine-tuning, test-time adaptation, or target-dataset-dependent scale alignment.

#### 3.1.3. Evaluation Protocol and Metrics

For evaluation, dataset-specific depth-scale alignment procedures were adopted. For NYU-Depth V2, per-image least-squares scale-and-shift alignment was performed over valid pixels, followed by the standard Eigen crop. For KITTI, evaluation was conducted on the Eigen test split using the Garg crop, per-image median scaling, and a maximum depth of 80 m.

In this study, evaluation metrics consistent with previous works [9,10,11,12,13,14,15,16,17,18,19,20,21,22,23,24,25] are adopted to assess prediction accuracy, including Root Mean Square Error (RMSE), Absolute Relative Error (AbsRel), Squared Relative Error (SqRel), and Accuracy (Acc). Let ${\hat{d}}_{i}$ denote the predicted depth and $d_{i}$ the ground-truth depth, with N valid pixels in an image. The definitions of these metrics are given in Equations (14)–(17).

(14) $$RMSE=\sqrt{\frac{1}{N}\sum_{i=1}^{N}({\hat{d}}_{i}-d_{i}{)}^{2}}$$

(15) $$AbsRel=\frac{1}{N}\sum_{i=1}^{N}\frac{|{\hat{d}}_{i}-{\;d}_{i}|}{d_{i}}$$

(16) $$SqRel=\frac{1}{N}\sum_{i=1}^{N}\frac{\left({\hat{d}}_{i}\;-\;d_{i}{)}^{2}\right.}{d_{i}}$$

(17) $$\left\{\begin{array}{c} \delta_{i}=max\left(\frac{{\hat{d}}_{i}}{d_{i}},\frac{d_{i}}{{\hat{d}}_{i}}\right)\\ {Acc}_{1}=\frac{1}{N}\sum_{i=1}^{N}I\left(\delta_{i}<1.25\right)\\ \begin{array}{c} {Acc}_{2}=\frac{1}{N}\sum_{i=1}^{N}I\left(\delta_{i}<1.25^{2}\right)\\ {Acc}_{3}=\frac{1}{N}\sum_{i=1}^{N}I\left(\delta_{i}<1.25^{3}\right) \end{array} \end{array}\right.$$

In addition to accuracy evaluation, this study further assesses model performance in terms of computational complexity and inference efficiency. Specifically, floating-point operations (FLOPs) are used to measure the theoretical complexity of the model, while frames per second (FPS) and single-frame inference time (Latency) are adopted to evaluate its practical inference efficiency. Here, FLOPs represent the theoretical computational cost under a given input resolution, reflecting the model complexity, whereas FPS and Latency are used to characterize the runtime efficiency in real-world deployment scenarios.

### 3.2. Ablation Study

#### 3.2.1. Progressive Ablation Study

To examine the progressive construction of LapCR-Net, a series of ablation experiments were conducted on the NYU-Depth V2 dataset under identical training and evaluation settings. The baseline model (Exp. 1) consists of a vanilla MobileNetV3 encoder and a conventional U-Net decoder. The FA-Mobile module and the bottleneck global-context module were then progressively introduced in Exps. 2–3. The individual and collaborative effects of SFR and DCM were evaluated in Exps. 4–6, followed by a comparison between conventional U-Net decoding and the proposed Laplacian residual reconstruction mechanism in Exps. 7–8. Finally, Guided Refine was incorporated to form the complete LapCR-Net. The model configurations and quantitative results are presented in Table 1 and Table 2, respectively.

As shown in Exps. 1–3, progressively introducing FA-Mobile and bottleneck-based global context modeling reduces RMSE from 0.4113 to 0.3785, demonstrating the effectiveness of enhanced multi-scale feature representation and global semantic modeling. When SFR or DCM is introduced individually in Exps. 4–5, only moderate improvements are observed. In contrast, their joint integration in Exp. 6 produces more consistent gains across the evaluation metrics, indicating that structure-aware feature recalibration and depth-guided residual prediction are complementary.

The results of Exps. 7–8 further demonstrate the benefit of replacing conventional U-Net decoding with progressive Laplacian residual reconstruction. Incorporating Guided Refine into the final model further reduces RMSE from 0.3685 to 0.3654 and SqRel from 0.0614 to 0.0599 while increasing the parameter count by only 0.04M. Overall, the progressive ablation results verify that the proposed components contribute complementary improvements and collectively enable LapCR-Net to achieve the best overall performance.

#### 3.2.2. Independent Component-Wise Ablation Study

The progressive ablation study introduces multiple components sequentially and therefore cannot fully isolate their individual contributions under the same complete-model configuration. To address this issue, an additional independent component-wise ablation study was conducted. Since the effects of SFR, DCM, and Guided Refine have already been evaluated in Exps. 4–8, the present study focuses on independently removing FA-Mobile, the TCA bottleneck, Global Gate, the uncertainty-aware loss, and the edge-aware smoothness loss. All other network components, training settings, data preprocessing procedures, hyperparameters, and evaluation protocols were kept unchanged. The detailed experimental configurations and quantitative results are presented in Table 3 and Table 4, respectively.

As shown in Table 4, removing any individual module or loss term leads to a decline in performance. After removing FA-Mobile, RMSE increases from 0.3654 to 0.3723, while Acc1 decreases from 89.3% to 88.8%, indicating that the interaction between local structural information and global semantic information contributes to effective multi-scale feature representation. Removing the TCA bottleneck increases RMSE to 0.3741, representing the most pronounced degradation among the structural-module ablations and demonstrating the importance of long-range dependency modeling for suppressing large depth errors and maintaining global geometric consistency. The effect of Global Gate is relatively smaller, but it still provides complementary global feature recalibration. Regarding the loss functions, removing the uncertainty-aware loss increases RMSE, AbsRel, and SqRel to 0.3743, 0.1069, and 0.0647, respectively, indicating that reliability-based error weighting helps the model handle ambiguous and challenging regions. Removing the edge-aware smoothness loss also degrades performance, confirming its contribution to preserving local structural consistency and depth transitions.

Combined with the progressive ablation results in Table 1 and Table 2, these findings show that the proposed components address different aspects of the task. SFR and DCM enhance the interaction between structural and depth information, Laplacian residual reconstruction improves coarse-to-fine depth recovery, Guided Refine strengthens final boundary refinement, and the remaining modules and loss terms further improve feature representation, global consistency, and optimization stability. Therefore, the complete model achieves the best overall performance through the coordinated contributions of all components.

### 3.3. Quantitative Results

#### 3.3.1. Overall Depth Estimation Performance

To further quantitatively evaluate the reliability and effectiveness of LapCR-Net, comprehensive comparative experiments were conducted on the NYU-Depth V2 and KITTI datasets under two complementary settings.

For the controlled quantitative comparison, LapDepth, GuideDepth, and DaNet were selected as representative supervised monocular depth estimation methods and were retrained together with LapCR-Net according to the unified training protocol described in Section 3.1.1. These methods represent different technical directions, including Laplacian residual reconstruction, lightweight guided decoding, and global depth modeling. All methods used the same training, validation, and test splits, and no pretrained depth-estimation weights were used during training. Where officially available, the encoders were initialized with ImageNet-pretrained weights; specifically, LapDepth employed its official ResNeXt101 encoder. The original network architectures and method-specific loss functions were retained for all compared methods. All retrained models were quantitatively evaluated using the unified evaluation protocol described in Section 3.1.3, and the results are reported in Table 5.

In addition to the controlled experiments, HR-Depth, LiteMono, Depth Anything, and BoRe-Depth were included as extended comparison methods. Their training paradigms differ substantially from the supervised setting adopted in this work. Specifically, HR-Depth, LiteMono, and BoRe-Depth primarily employ self-supervised learning based on monocular video sequences, photometric reconstruction, or pseudo-depth constraints, whereas Depth Anything relies on large-scale external data and pretraining strategies. Therefore, their officially released pretrained checkpoints were retained and re-evaluated using the unified evaluation protocol and evaluation scripts described in Section 3.1.3. These experiments further assess the overall performance and competitiveness of LapCR-Net against representative methods developed under different supervision paradigms and pretraining strategies, and the results are reported in Table 6.

The results on NYU-Depth V2 demonstrate the effectiveness of LapCR-Net under both controlled and extended comparison settings. As shown in Table 5, LapCR-Net achieves the best performance across all reported metrics among the models retrained under identical conditions, with an RMSE of 0.3654, an AbsRel of 0.1032, and an SqRel of 0.0599. Compared with LapDepth, GuideDepth, and DaNet, the proposed method provides consistently lower prediction errors while using only 5.4M parameters. In particular, LapCR-Net substantially outperforms LapDepth despite using fewer than one-tenth of its parameters, demonstrating the effectiveness and parameter efficiency of the proposed structure-aware and depth-guided Laplacian reconstruction mechanism. The extended comparison in Table 6 further shows that LapCR-Net achieves the lowest RMSE and SqRel among the listed pretrained models. Although Depth Anything obtains a lower AbsRel and a higher Acc1, it uses a substantially larger model and benefits from large-scale external pretraining. LapCR-Net nevertheless achieves comparable Acc2 and Acc3 values with a considerably smaller parameter count.

LapCR-Net demonstrates more significant performance improvements on the KITTI dataset. It achieves the best results across key metrics, including AbsRel, SqRel, and Acc1, with Acc1 reaching 93.5%, significantly outperforming other competing methods. Meanwhile, SqRel is reduced to 0.323, indicating improved error control in scenarios with large depth variations and long-range distances. In addition, LapCR-Net achieves substantially lower RMSE than lightweight methods such as GuideDepth and BoRe-Depth, and even surpasses several large-scale models. All accuracy metrics (Acc1, Acc2, and Acc3) reach competitive levels among the compared methods, further demonstrating the superiority of the proposed approach in terms of overall depth prediction accuracy and stability.

#### 3.3.2. Boundary-Region Evaluation and Sensitivity Analysis

To further assess the ability of LapCR-Net to recover depth discontinuities and object boundaries, we conducted an additional boundary-region evaluation on the NYU-Depth V2 dataset. Owing to its dense ground-truth depth maps, NYU-Depth V2 provides a more reliable basis for boundary-aware quantitative analysis than KITTI. Boundary candidates were extracted from the Sobel gradient magnitudes of the ground-truth depth maps, and local boundary regions were subsequently generated through morphological dilation. Boundary RMSE and Boundary AbsRel were calculated exclusively over the valid pixels within the resulting boundary masks.

Because the definition of the boundary mask may affect the absolute values of the boundary-region metrics, we further evaluated the sensitivity of the results to the Sobel gradient threshold and dilation kernel size. Specifically, the Sobel percentile threshold was varied among the 85th, 90th, and 95th percentiles, while the dilation kernel size was varied among 1 × 1, 3 × 3, and 5 × 5. The 1 × 1 setting indicates that no additional morphological dilation was applied. It should be emphasized that these parameters were used only to construct the boundary masks for quantitative evaluation and were not involved in model training or inference. The boundary-region comparison and parameter sensitivity results are summarized in Table 7.

As shown in Table 7, increasing the Sobel percentile threshold concentrates the evaluation on sharper depth discontinuities, resulting in higher boundary errors for all methods. Nevertheless, LapCR-Net consistently achieves the lowest Boundary RMSE at the 85th, 90th, and 95th percentiles, reducing it by approximately 2.2%, 4.1%, and 4.0%, respectively, compared with Depth Anything. For Boundary AbsRel, LapCR-Net performs best at the 85th percentile, while Depth Anything is marginally better at the higher thresholds. These results demonstrate that LapCR-Net provides more reliable absolute depth reconstruction around increasingly challenging boundary regions.

Increasing the dilation size expands the evaluated boundary region and generally reduces the boundary errors. Under all three dilation settings, LapCR-Net achieves the lowest Boundary RMSE and Boundary AbsRel, with Boundary RMSE reductions of approximately 6.6%, 8.9%, and 2.2% over Depth Anything for the 1 × 1, 3 × 3, and 5 × 5 settings, respectively. This consistent advantage confirms the robustness of LapCR-Net across boundary regions of different widths.

Overall, these results verify that the boundary reconstruction performance of LapCR-Net remains stable across different Sobel thresholds and dilation sizes, demonstrating the effectiveness of the proposed Laplacian residual reconstruction framework in preserving depth discontinuities and object boundaries.

### 3.4. Qualitative Results

**Figure 8:**
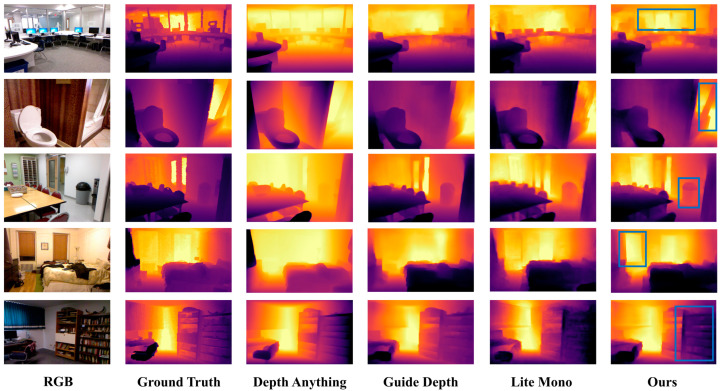
Qualitative Results on the NYU-Depth V2 Dataset. Blue boxes indicate highlighted regions for detailed comparison, and colors represent the predicted depth values.

Figure 8 presents a qualitative comparison of different methods on the NYU-Depth V2 dataset. In office and laboratory scenes (Rows 1 and 3), the proposed model accurately reconstructs regular geometric structures, such as monitors, window frames, and printers, with clear structural transitions and continuous depth variations. In cluttered scenes (Row 5), it maintains stable depth discrimination for slender structures and occluded regions. In multi-furniture environments (Row 4), the model effectively captures depth discontinuities of key structures, such as headboards and windows. Moreover, in challenging scenarios with significant illumination variations or strong local shadows, such as bathrooms and restrooms (Row 2), the model preserves consistent depth predictions across regions with varying brightness levels. These results demonstrate that LapCR-Net exhibits strong stability and robust generalization capability in complex indoor environments.

**Figure 9:**
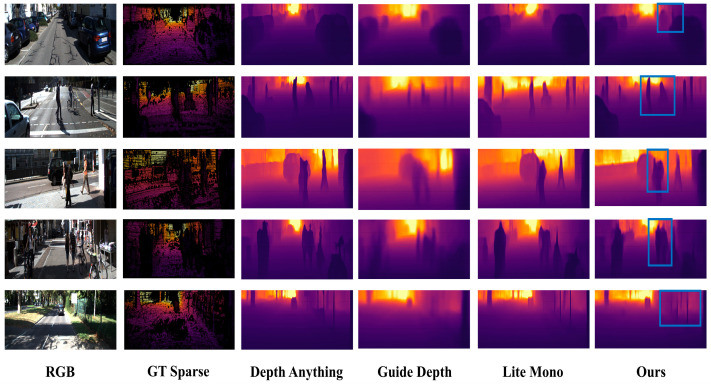
Qualitative Results on the KITTI Dataset. Blue boxes indicate highlighted regions for detailed comparison, and colors represent the predicted depth values.

Figure 9 presents qualitative comparisons of different methods on the KITTI dataset together with the corresponding sparse ground-truth depth observations. In scenes with pronounced shadows (Row 1), LapCR-Net maintains coherent depth predictions for pedestrians and surrounding objects under challenging illumination conditions. In wide and well-lit scenes (Rows 2–4), LapCR-Net generally preserves the overall integrity of pedestrian contours and maintains smooth depth transitions across the road scene. Although Depth Anything produces visually sharper boundaries in some cases, the proposed method yields depth structures that are generally consistent with the sparse ground-truth observations while using substantially fewer parameters. In long-range and sparse-structure scenarios (Row 5), LapCR-Net remains capable of preserving the major scene geometry and depth hierarchy. Overall, the qualitative results indicate that LapCR-Net achieves a favorable balance between contour integrity, depth continuity, structural consistency, and model efficiency in complex outdoor environments.

**Figure 10:**
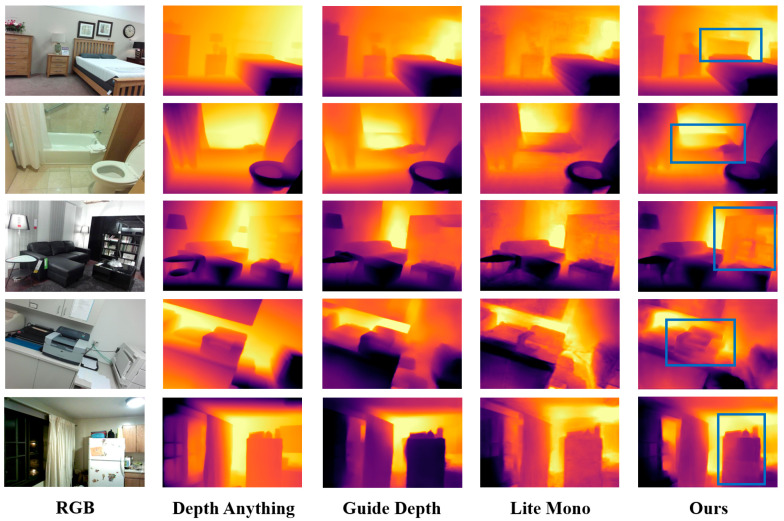
Zero-shot qualitative comparison on the SUN RGB-D dataset. Blue boxes indicate highlighted regions for detailed comparison, and colors represent the predicted depth values.

To further evaluate the cross-dataset generalization capability of LapCR-Net, Figure 10 presents a zero-shot generalization comparison on the SUN RGB-D dataset. None of the methods involved fine-tuning, test-time adaptation, or target-dataset-dependent scale alignment. The same input resizing, depth-scaling procedure, and visualization settings were applied to all methods. As can be observed, Depth Anything generally produces smooth depth distributions but tends to weaken local depth variations between adjacent structures. GuideDepth and Lite-Mono preserve the overall scene layout reasonably well, although local blurring and structural distortions remain visible in several challenging regions. In comparison, LapCR-Net generates more coherent depth structures and clearer local depth discontinuities around the bed, bathtub, shelving unit, printer, and refrigerator, as highlighted by the blue boxes. These results indicate that the proposed progressive Laplacian residual reconstruction strategy remains effective in unseen indoor environments and exhibits strong zero-shot cross-dataset generalization capability.

### 3.5. Model Efficiency, Complexity, and Embedded Deployment Analysis

#### 3.5.1. Desktop GPU Efficiency and Complexity

To evaluate the efficiency and computational complexity of LapCR-Net, we compared it with HR-Depth, GuideDepth, and Lite-Mono under the same experimental setting. All models were tested on an NVIDIA RTX 3090 GPU with an input resolution of 640×192, a batch size of 1, 50 warm-up iterations, and 300 test iterations. Only forward inference time was measured, excluding image loading, preprocessing, result saving, and visualization. No TensorRT optimization or mixed-precision inference was applied. The results are presented in Table 8.

As shown in Table 8, LapCR-Net requires 5.551 G FLOPs, which is only slightly higher than GuideDepth but substantially lower than Lite-Mono and HR-Depth. It achieves 110 FPS with a latency of 9.09 ms, satisfying real-time inference requirements. Although its speed is lower than that of the comparison methods, runtime performance is not determined solely by FLOPs or parameter count. HR-Depth mainly relies on standard convolutional operations that are highly optimized by cuDNN, while GuideDepth benefits from an efficient lightweight decoding strategy. In contrast, the progressive Laplacian residual reconstruction and SFR module in LapCR-Net introduce additional cross-scale fusion and refinement operations, improving depth estimation accuracy at the cost of moderate runtime overhead. Overall, LapCR-Net achieves the best estimation accuracy while maintaining a compact model size, low computational complexity, and real-time performance, demonstrating a favorable trade-off between accuracy and efficiency.

#### 3.5.2. Embedded Deployment Evaluation

To further evaluate the practical deployment capability of LapCR-Net under resource-constrained conditions, the model was deployed on a relatively low-specification NVIDIA Jetson Orin NX 8 GB embedded platform with limited computing and memory resources, operating under a fixed 15 W power mode. The platform was configured with JetPack 6.2.2, CUDA 12.6, and PyTorch 2.5.0. All experiments were conducted using FP32 precision, a batch size of 1, and an input resolution of 640×192, while the remaining test settings were kept consistent with those used in the desktop GPU evaluation. During inference, system memory usage and module power consumption were monitored using the built-in tegrastats utility with a sampling interval of 100 ms. Peak system memory was obtained from the RAM usage recorded during continuous inference, while average module power was calculated from the instantaneous VDD_IN measurements collected during periods of stable high GPU utilization.

As shown in Table 9, all methods exhibit relatively limited inference speeds on the low-specification Jetson Orin NX 8 GB platform under the 15 W power mode, mainly due to the restricted computing and memory resources of the device. Nevertheless, LapCR-Net achieves 13.7 FPS with a latency of 72.0 ms while requiring only 587.95 MB of peak system memory and 11.25 W of average power. Its runtime is faster than HR-Depth and comparable to Lite-Mono, while its memory usage is nearly the lowest among the compared methods. These results demonstrate that LapCR-Net can be stably deployed even on a highly resource-constrained embedded platform. Therefore, more capable edge devices with larger memory capacity and greater computing resources are expected to provide more favorable deployment performance.

## 4. Discussion

### 4.1. Specific Benefits and Comparative Interpretation

The experimental results indicate that the main benefit of LapCR-Net lies in achieving reliable depth reconstruction with a compact architecture rather than uniformly outperforming all competing methods across every evaluation metric. As demonstrated by the multi-model quantitative comparisons in Table 5 and Table 6, together with the boundary-region evaluation and sensitivity analysis presented in Table 7, LapCR-Net maintains competitive global depth accuracy while exhibiting more pronounced advantages in boundary-sensitive regions. These findings indicate that the proposed model effectively utilizes its limited representational capacity to preserve structural consistency, recover depth discontinuities, and progressively correct cross-scale prediction errors.

Compared with the larger Depth Anything model, LapCR-Net achieves a more favorable balance between model compactness and prediction quality. Although Depth Anything retains advantages in certain accuracy metrics, LapCR-Net delivers comparable overall performance with substantially fewer parameters. Its practical value therefore lies not only in reducing model size, but also in preserving reliable geometric information under lightweight computational constraints.

This performance can be attributed to the coordinated design of the encoder and decoder. In the encoder, the FA-Mobile module strengthens the interaction between local structural details and global semantic information, while the TCA and Global Gate modules enhance long-range geometric modeling. In the decoder, the SFR module improves the geometric discriminability of the encoded features, and the DCM introduces the reconstructed coarse depth as an explicit spatial prior for residual prediction. Through this collaborative structure–depth modeling process, LapCR-Net progressively refines local geometric details while maintaining the global scene layout.

Accordingly, the specific benefit of LapCR-Net can be summarized as parameter-efficient, structure-aware depth reconstruction. The method is particularly suitable for applications that simultaneously require a compact model, real-time inference, and reliable preservation of object boundaries and depth discontinuities.

### 4.2. Analysis of the Effect of Laplacian Residual Reconstruction

Compared with the conventional U-Net-style decoding structure, the proposed Laplacian residual reconstruction mechanism demonstrates clear advantages in structural recovery and error control. As demonstrated by the ablation results in Table 2, when feature fusion relies solely on conventional skip connections, the model remains susceptible to over-smoothing in detailed regions. In contrast, after introducing the Laplacian residual reconstruction mechanism, the depth prediction at each scale is progressively refined based on the upsampled depth from the preceding scale, enabling more accurate representation of depth discontinuities and object boundaries. The consistent improvements observed in the ablation experiments confirm that progressive residual reconstruction provides more effective structural refinement than conventional direct upsampling and feature fusion.

From a theoretical perspective, Laplacian decomposition represents a signal as the combination of a low-frequency base and high-frequency residuals. This formulation shifts the learning focus from directly fitting the full depth distribution to modeling localized residual components that are easier to optimize. As a result, the variance of the learning target is reduced to some extent, leading to improved optimization stability. Moreover, under the guidance of the DCM module, the residual generation process explicitly leverages the depth from the previous scale as a spatial constraint, facilitating more stable cross-scale information propagation and effectively suppressing error accumulation during progressive reconstruction.

Overall, this mechanism not only improves depth prediction accuracy but also enhances the model’s ability to represent complex structural regions, making it a key factor contributing to the performance gains of the proposed method.

### 4.3. Trade-Off Among Accuracy, Efficiency, and Embedded Deployability

The results in Table 8 and Table 9 provide a comprehensive evaluation of LapCR-Net in terms of prediction accuracy, computational efficiency, and practical deployment capability. On the NVIDIA RTX 3090, LapCR-Net contains only 5.4M parameters and requires 5.551 GFLOPs, achieving an inference speed of 110 FPS with a latency of 9.09 ms. Although its inference speed is lower than that of HR-Depth, GuideDepth, and Lite-Mono, it achieves the best overall depth-estimation accuracy among the compared lightweight models. This indicates that the additional computational operations introduced by LapCR-Net primarily contribute to improved reconstruction quality rather than maximizing inference throughput.

The difference in inference speed mainly arises from the progressive Laplacian residual reconstruction process and the multi-branch feature interaction design. Specifically, the sequential coarse-to-fine residual prediction, cross-scale feature fusion, and structure-aware refinement operations introduce additional computational and memory-access overhead. However, these components also enhance structural representation, boundary reconstruction, and prediction stability. Moreover, parameter count and FLOPs cannot fully characterize actual runtime efficiency, which is also affected by operator type, feature-map transformation, memory access, and hardware optimization. For example, HR-Depth mainly relies on standard convolutional operations that are efficiently supported by cuDNN, whereas GuideDepth adopts a relatively simple lightweight decoding strategy. Therefore, the desktop GPU results reflect a reasonable trade-off in which moderate runtime overhead is exchanged for improved depth-estimation accuracy and structural reliability, while real-time performance is maintained.

The embedded evaluation further demonstrates that model efficiency should not be assessed solely on desktop GPUs, but should also be examined under actual resource constraints. Under the fixed 15 W power mode of the NVIDIA Jetson Orin NX 8 GB, LapCR-Net achieves 13.7 FPS with a latency of 72.0 ms, a peak system memory usage of 587.95 MB, and an average power consumption of 11.25 W. Its inference speed is higher than that of HR-Depth and comparable to that of Lite-Mono, while its peak memory usage is nearly the lowest among the compared models. These results indicate that the compact architecture of LapCR-Net is well suited to embedded platforms with limited memory, computing resources, and power budgets.

Overall, the principal advantage of LapCR-Net does not lie in achieving the highest inference speed, but in delivering high depth-estimation accuracy with a compact model and low memory demand. Therefore, the proposed method is particularly suitable for resource-constrained visual perception tasks that require a balanced consideration of prediction accuracy, structural reliability, memory usage, and deployment cost.

### 4.4. Favorable Scene Types and Application Scope

To investigate whether the relative performance of LapCR-Net varies across different types of scenes, we conducted a scene-attribute stratified evaluation on the NYU-Depth V2 test set. Because the dataset provides dense ground-truth depth maps, the structural complexity of each test image was quantified using the mean gradient magnitude of logarithmic depth. The 654 test images were then ranked according to their structural-complexity scores and equally divided into low-, medium-, and high-complexity groups, with 218 images in each group. GuideDepth, Lite-Mono, Depth Anything, and LapCR-Net were evaluated using the same input preprocessing, scale-and-shift alignment, and metric-computation protocol. The corresponding results are presented in Table 10.

The stratified results reveal a clear complexity-dependent performance trend. In the low- and medium-complexity groups, Depth Anything achieves the best overall accuracy, while LapCR-Net remains highly competitive in terms of RMSE, with differences of only 0.0017 and 0.0038, respectively. As scene complexity increases, however, the advantage of LapCR-Net becomes more evident. In the high-complexity group, LapCR-Net achieves the best RMSE and AbsRel values of 0.5619 and 0.1287, outperforming Depth Anything by 3.9% and 16.6%, respectively. It also substantially surpasses GuideDepth and Lite-Mono in this group. These results indicate that the relative benefit of LapCR-Net increases with structural complexity. In particular, its progressive Laplacian residual reconstruction mechanism is more effective in scenes containing frequent depth transitions and complex geometric structures, whereas its advantage is less pronounced in structurally simple scenes. Therefore, LapCR-Net is particularly suitable for depth-estimation applications involving complex indoor environments and pronounced geometric variations.

### 4.5. Limitations and Future Directions

Although LapCR-Net achieves a favorable balance among depth accuracy, structural reconstruction, and model efficiency, several limitations remain. Its relative advantage is less pronounced in scenes dominated by large homogeneous regions with weak geometric variation, where limited high-frequency information is available for residual reconstruction. Moreover, the current stratified evaluation characterizes images according to geometric depth complexity rather than semantic scene or object categories. Further category-specific analysis would therefore require datasets with consistent scene-level and object-level annotations.

LapCR-Net is currently designed for individual static RGB images and does not explicitly exploit temporal relationships in dynamic data. Studies from other disciplines have demonstrated that temporal correlations and evolving motion patterns contain valuable information for feature identification and prediction. Meyer et al. [37] combined statistical and machine learning methods to analyze long-term springbok trajectories and showed that movement predictability is related to directional persistence and daily activity cycles. Similarly, Luxem et al. [38] proposed a deep variational framework for identifying hierarchical behavioral motifs from spatiotemporal animal-motion data. In video depth estimation, independently processing consecutive frames may produce temporal flickering and inconsistent geometric structures. Video Depth Anything addresses this problem through explicit spatial–temporal feature modeling and a temporal consistency constraint [39].

Accordingly, extending LapCR-Net from spatial Laplacian residual reconstruction to spatial–temporal residual modeling represents an important future direction. Reconstructed depth and motion-aligned features from previous frames could serve as temporal priors for current-frame residual prediction, while uncertainty-aware temporal fusion could suppress unreliable information caused by moving objects, occlusions, or rapid camera motion. Future work will focus on lightweight temporal feature interaction and cross-frame residual refinement to improve video-depth consistency while preserving the computational efficiency required for embedded deployment.

## 5. Conclusions

This paper proposes LapCR-Net, a lightweight monocular depth estimation framework based on progressive Laplacian residual reconstruction. The novelty of the proposed framework lies in jointly addressing cross-scale inconsistency and structural over-smoothing within a unified reconstruction framework. Instead of directly regressing dense depth maps, LapCR-Net decomposes depth recovery into low-frequency structure prediction and high-frequency residual correction through the collaborative optimization of structure-aware feature recalibration, depth-guided residual learning, and uncertainty-aware refinement. Specifically, the proposed LapCR-Encoder enhances multi-scale semantic and geometric representation through collaborative feature modeling, while the LapCR-Decoder progressively reconstructs depth structures via residual-coupled Laplacian reconstruction. Together with uncertainty-guided adaptive residual refinement, the proposed framework effectively improves structural consistency, boundary preservation, and structural detail preservation under lightweight computational constraints.

Experimental results on the NYU-Depth V2 and KITTI benchmarks demonstrate that LapCR-Net achieves competitive depth-estimation performance with only 5.4M parameters, providing a favorable balance among prediction accuracy, structural detail preservation, computational efficiency, and deployment cost. The boundary-region evaluation and scene-complexity-stratified analysis further show that the proposed framework is particularly effective in preserving depth discontinuities and complex geometric structures, with its relative advantage becoming more evident in high-complexity scenes. In addition, the desktop and embedded evaluations confirm that LapCR-Net maintains practical inference efficiency and low memory demand, making it suitable for resource-constrained visual-perception applications requiring structurally reliable depth estimation.

Nevertheless, LapCR-Net remains less advantageous in scenes dominated by large homogeneous regions with weak geometric variation, where limited high-frequency information is available for residual reconstruction. Moreover, the current framework is designed for individual static RGB images and does not explicitly exploit temporal relationships in dynamic data. Future work will therefore focus on extending spatial Laplacian residual reconstruction toward lightweight spatiotemporal residual modeling by incorporating motion-aligned temporal priors, uncertainty-aware temporal fusion, and cross-frame residual refinement. This extension is expected to improve temporal consistency in video depth estimation while preserving the compactness and computational efficiency of the proposed framework.

## Figures and Tables

**Table 1:** Configurations of the progressive ablation models.

Exp.	MobileNetV3	FA Module	Bottleneck	SFR	DCM	UNet Skip	Laplacian Reconstruction	Guided Refine
1	√	×	×	×	×	√	×	×
2	√	√	×	×	×	√	×	×
3	√	√	√	×	×	√	×	×
4	√	√	√	√	×	√	×	×
5	√	√	√	×	√	√	×	×
6	√	√	√	√	√	√	×	×
7	√	√	√	√	√	√	×	√
8	√	√	√	√	√	×	√	×
Ours	√	√	√	√	√	×	√	√

**Note:** “√” indicates the inclusion of the corresponding module, while “×” indicates its exclusion.

**Table 2:** Results of the progressive ablation study.

Exp.	RMSE ↓	AbsRel ↓	SqRel ↓	Acc1 ↑	Acc2 ↑	Acc3 ↑	Params/$10^{6}$
1	0.4113	0.1156	0.0740	87.1%	97.4%	99.4%	3.54
2	0.3812	0.1110	0.0716	88.5%	97.5%	99.4%	3.61
3	0.3785	0.1092	0.0638	89.0%	97.5%	99.4%	3.87
4	0.3769	0.1052	0.0643	89.1%	97.5%	99.4%	3.99
5	0.3753	0.1059	0.0641	89.1%	97.7%	99.4%	4.67
6	0.3721	0.1042	0.0635	89.3%	97.8%	99.5%	4.95
7	0.3703	0.1033	0.0616	89.3%	97.8%	99.5%	5.33
8	0.3685	0.1038	0.0614	89.2%	97.9%	99.5%	5.36
Ours	**0.3654**	**0.1032**	**0.0599**	**89.3%**	**97.9%**	**99.5%**	5.40

**Note:** Bold values indicate the best performance among all compared methods.

**Table 3:** Configurations of the independent component-wise ablation models.

Exp.	FA Module	Bottleneck	Global Gate	Uncertainty Loss	Smoothness Loss
1	×	√	√	√	√
2	√	×	√	√	√
3	√	√	×	√	√
4	√	√	√	×	√
5	√	√	√	√	×
Ours	√	√	√	√	√

**Note:** “√” indicates the inclusion of the corresponding module, while “×” indicates its exclusion.

**Table 4:** Results of the independent component-wise ablation study.

Exp.	RMSE ↓	AbsRel ↓	SqRel ↓	Acc1 ↑	Acc2 ↑	Acc3 ↑	Params/$10^{6}$
1	0.3723	0.1060	0.0640	88.8%	97.7%	99.4%	5.35
2	0.3741	0.1040	0.0617	89.2%	97.7%	99.4%	5.16
3	0.3676	0.1047	0.0610	89.2%	97.9%	99.5%	5.39
4	0.3743	0.1069	0.0647	89.0%	97.6%	99.4%	5.39
5	0.3691	0.1045	0.0609	89.2%	97.9%	99.5%	5.39
Ours	**0.3654**	**0.1032**	**0.0599**	**89.3%**	**97.9%**	**99.5%**	5.40

**Table 5:** Quantitative comparison results under identical training and evaluation conditions.

Dataset	Method	Params/$10^{6}$	RMSE ↓	AbsRel ↓	SqRel ↓	Acc1 ↑	Acc2 ↑	Acc3 ↑
NYU Depth V2	LapDepth	73.5	0.4410	0.1189	0.0860	89.2%	97.1%	99.2%
	GuideDepth	5.5	0.4435	0.1236	0.0791	86.4%	97.2%	99.3%
	DaNet	7.8	0.5101	0.1475	0.0836	82.5%	95.3%	99.0%
	Ours	5.4	**0.3654**	**0.1032**	**0.0599**	89.3%	**97.9%**	**99.5%**
	LapDepth	73.5	3.885	0.091	0.552	88.1%	96.8%	99.1%
KITTI	GuideDepth	5.5	4.427	0.119	0.738	86.8%	96.7%	99.1%
	DaNet	7.8	6.115	0.134	0.753	84.5%	96.1%	99.0%
	Ours	5.4	**3.050**	**0.080**	**0.323**	**93.5%**	**99.0%**	**99.7%**

**Table 6:** Comparison with official pretrained models.

Dataset	Method	Params/$10^{6}$	RMSE ↓	AbsRel ↓	SqRel ↓	Acc1 ↑	Acc2 ↑	Acc3 ↑
NYU Depth V2	LiteMono	8.1	0.4570	0.1180	0.0730	87.8%	96.3%	98.8%
	Depth Anything	24.8	0.3711	**0.0995**	0.0608	**91.7%**	97.8%	99.5%
	BoRe-Depth	8.7	0.4290	0.1110	0.0635	88.3%	97.1%	99.3%
	Ours	5.4	**0.3654**	0.1032	**0.0599**	89.3%	**97.9%**	**99.5%**
	HrDepth	14.6	3.538	0.083	0.455	90.5%	97.6%	99.2%
	LiteMono	8.1	3.793	0.081	0.465	91.8%	98.2%	99.6%
KITTI	Depth Anything	24.8	**2.438**	0.100	0.421	90.6%	97.5%	99.5%
	BoRe-Depth	8.7	4.323	0.103	0.647	88.9%	96.7%	98.6%
	Ours	5.4	3.050	**0.080**	**0.323**	**93.5%**	**99.0%**	**99.7%**

**Table 7:** Sensitivity analysis of boundary-region evaluation on the NYU-Depth V2 dataset.

Parameter Setting	GuideDepth	LiteMono	Depth Anything	Ours
**Sobel threshold sensitivity (**5×5 **dilation)**				
85th percentile	0.5404/0.1263	0.8459/0.2273	0.4630/0.1193	**0.4527** **/0.1138**
90th percentile	0.5658/0.1337	0.8735/0.2354	0.5014/**0.1211**	**0.4808**/0.1218
95th percentile	0.6034/0.1461	0.9024/0.2429	0.5495/0.1274	**0.5277** **/0.1357**
**Dilation-size sensitivity (85th-percentile threshold)**				
1 × 1	0.5911/0.1348	0.8777/0.2255	0.5380/0.1259	**0.5023** **/0.1213**
3 × 3	0.5620/0.1294	0.8603/0.2265	0.5189/0.1225	**0.4728** **/0.1163**
5 × 5	0.5404/0.1263	0.8459/0.2273	0.4630/0.1193	**0.4527/0.1138**

**Note:** Each entry reports Boundary RMSE/Boundary AbsRel. Lower values indicate better performance. For the Sobel-threshold analysis, the dilation kernel was fixed at 5 × 5; for the dilation-size analysis, the Sobel threshold was fixed at the 85th percentile. A 1 × 1 kernel indicates no additional morphological dilation.

**Table 8:** Model efficiency and complexity on NVIDIA RTX 3090.

Method	Params/$10^{6}$	FlOPs/G	FPS ↑	Latency/ms ↓	AbsRel ↓	RMSE ↓
HrDepth	14.6	31.624	135	7.40	0.083	3.538
GuideDepth	5.82	4.237	153	6.53	0.114	4.35
LiteMono	8.1	11.212	124	8.06	0.081	3.793
Ours	5.4	5.551	110	9.09	0.080	3.050

**Table 9:** Embedded performance of LapCR-Net on NVIDIA Jetson Orin NX 8 GB.

Method	Params/$10^{6}$	FPS ↑	Latency/ms ↓	Peak System Memory (MB) ↓	Average Power (W) ↓
HrDepth	14.6	12.5	80.0	667.10	11.10
GuideDepth	5.82	21.1	47.4	587.40	10.73
LiteMono	8.1	14.5	68.9	643.36	11.75
Ours	5.4	13.7	72.0	587.95	11.25

**Table 10:** Performance comparison across different levels of scene structural complexity.

Method	Low Complexity		Medium Complexity		High Complexity	
	RMSE ↓	AbsRel ↓	RMSE ↓	AbsRel ↓	RMSE ↓	AbsRel ↓
GuideDepth	0.2195	0.0760	0.3487	0.0911	0.7625	0.1897
LiteMono	0.2150	0.0813	0.3519	0.0988	0.8036	0.1739
Depth Anything	**0.2013**	**0.0581**	**0.3275**	**0.0860**	0.5845	0.1544
Ours	0.2030	0.0829	0.3313	0.0980	**0.** **5619**	**0.1287**

## Data Availability

The datasets used in this study are publicly available. NYU-Depth V2 (https://cs.nyu.edu/~silberman/datasets/nyu_depth_v2.html (accessed on 31 July 2026)), KITTI (http://www.cvlibs.net/datasets/kitti/ (accessed on 31 July 2026)), and SUN RGB-D (https://rgbd.cs.princeton.edu/ (accessed on 31 July 2026)) datasets can be accessed from their respective public repositories.
